# Systematic Review: Proteomics-Driven Multi-Omics Integration for Alzheimer’s Disease Pathology and Precision Medicine

**DOI:** 10.3390/neurolint17120197

**Published:** 2025-12-02

**Authors:** Jonathan Mingsong Dong, Huan Zhong

**Affiliations:** 1 Lower Canada College, Montreal, QC H4A 2L1, Canada; jondong2008@gmail.com; 2 Michael Smith Laboratories, Department of Biochemistry and Molecular Biology, Life Sciences Institute, University of British Columbia, Vancouver, BC V6T 1Z3, Canada

**Keywords:** multi-omics, proteomics, Alzheimer’s disease, machine learning

## Abstract

Background: Neurodegenerative diseases remain a central topic in biomedical research, with Alzheimer’s disease (AD) being the most extensively studied. Recent advances in multi-omics integration, particularly proteomics-based approaches, have enabled a deeper understanding of AD-related molecular pathways and their interconnections. However, challenges such as data heterogeneity and the complexity of large-scale datasets continue to hinder comprehensive integration and model interpretation. Methods: A total of 792 publications were retrieved from PubMed, among which, 27 peer-reviewed studies from 2024 and 2025 focusing on proteomics-anchored multi-omics integration for AD were selected for detailed analysis. These papers were categorized based on their integration strategies, omics combinations, and analytical methodologies. Additionally, statistical analysis of 218 studies published in 2024–2025 was performed to identify dominant omics layers and common integration trends. Results: Proteomics emerged as the most frequently studied omics layer and was most often integrated with transcriptomics in AD multi-omics studies. The analysis also revealed recurrent machine learning methods used for feature extraction and integration, along with key biological pathways implicated in AD pathogenesis, including amyloid metabolism, synaptic function, and neuroinflammation. Conclusions: This review provides a systematic overview of recent trends in proteomics-based multi-omics integration for AD research. It highlights both the scientific advances and methodological limitations in current approaches, serving as a valuable reference for researchers seeking to refine analytical frameworks and design more interpretable, data-driven studies in neurodegenerative disease research.

## 1. Introduction

Alzheimer’s disease (AD) involves molecular programs that are only partly captured by transcripts. Because proteins are the proximate effectors of cellular function—and because transcript–protein coupling in AD is often weak due to post-transcriptional regulation, post-translational modifications, degradation/aggregation, and shifts in cell-type composition—proteomics provides phenotype-proximal readouts of pathway activity. When embedded within a multi-omics framework, proteomics connects genetic susceptibility and RNA programs to functional protein modules and biomarker panels that extend beyond Aβ and tau. Accordingly, this review is organized around a proteomics-anchored multi-omics perspective.

Neurodegenerative diseases (NDs) are characterized by progressive neuronal loss in the brain, which disrupts synaptic connectivity essential for signal transmission and ultimately leads to dementia [1]. They include AD, Parkinson’s disease, amyotrophic lateral sclerosis, Huntington’s disease, and frontotemporal dementia, which are distinguished by distinct anatomical, molecular, and clinical hallmarks [2]. Among them, AD is the most prevalent, affecting an estimated 6.9 million and 7.2 million individuals aged ≥65 years in the United States in 2024 and 2025, respectively, and is defined as a slowly progressive disorder whose clinical manifestations worsen from mild or early symptoms to moderate and severe stages as pathology spreads across the cortex [3]. Large-scale studies have shown that transcript levels explain only part of the variance in protein abundance due to extensive post-transcriptional and translational control, and that proteomic networks in Alzheimer’s disease capture disease-related modules that are not visible at the RNA level [4].

Until now, no cure has been found to completely eliminate AD. Only treatments to slow the disease have been developed, like lecanemab (Leqembi^®^, Eisai Co., Ltd., Tokyo, Japan) and donanemab (Kisunla™, Eli Lilly and Company, Indianapolis, IN, USA) [3]. This is primarily because the causes of the disease are still not fully understood. Indeed, current hypotheses like the amyloid cascade hypothesis, which suggests that the accumulations of amyloid-beta cause neurodegeneration, and the tau hypothesis, which suggests that the accumulations of an abnormal form of the tau protein as neurofibrillary tangles cause neurodegeneration, are insufficient to explain AD pathology. In fact, numerous studies have shown the two hypotheses to be the consequences of earlier pathogenic processes, like metabolic dysfunction and inflammation [5]. More evidence is needed to establish causal relationships. For example, genetic evidence of AD pathology has been reported. Risk loci like the APOE ε4 allele and other genetic variants like amyloid precursor protein (APP), presenilin 1 (PSEN1), and presenilin 2 (PSEN2) variants have been shown to increase the risk of developing AD [6], while RNA transcripts, including mRNA, ncRNAs, circRNAs, miRNAs, piRNAs and siRNAs, have also been identified in the blood and cerebrospinal fluid in research on AD pathology [7].

Although the central dogma is often depicted as a linear gene–RNA–protein cascade, AD risk genes do not map straightforwardly onto measurable protein biomarkers. For example, the APOE gene encodes apolipoprotein E, which is routinely quantified as a fluid biomarker for AD, whereas other risk variants such as BIN1 and PICALM are not represented by proteins used in biofluid biomarker panels [8]. More broadly, post-transcriptional regulation, post-translational modifications (PTMs), protein degradation and aggregation, and shifts in cell-type composition can decouple RNA and protein abundance in AD, so that transcriptome–proteome relationships are highly context dependent rather than one-to-one [9].

Genomics surveys DNA variations with GWASs and WES/WGS, followed by fine-mapping and polygenic scoring to localize risk loci (e.g., APOE, TREM2) and infer causal genes and cell types [10]. Transcriptomics profiles RNA using bulk RNA-seq, single-cell/single-nucleus RNA-seq, and spatial assays to map pathways and splicing programs across brain cell populations [11,12]. Proteomics quantifies proteins via LC–MS/MS (DDA/DIA; SRM/PRM) and high-throughput affinity panels (Olink, Thermo Fisher Scientific, Waltham, MA, USA; SomaScan, Standard BioTools Inc., South San Francisco, CA, USA) in the brain, CSF, and plasma [13]. Applied to AD, these approaches reveal genetic susceptibility, microglial and synaptic expression changes, and protein signatures that extend beyond Aβ and tau toward inflammation, lipid metabolism, and neuronal resilience.

Among these layers, we foreground proteomics as the organizing lens of this review because proteins are the direct effectors of cellular function and are more closely tied to phenotypes [14]. Although few proteomic studies can be found in recent AD research—owing to challenges in sample preparation, dynamic range, and data integration [14]—when conducted, they often deliver unique, actionable readouts of pathway activity that genomics or transcriptomics alone cannot resolve [13,14,15].

Proteomics alone provides valuable information on dysregulated proteins in AD but is limited in explaining how these proteins are regulated or interconnected with genetic and transcriptomic variation. For instance, large-scale proteomic studies have identified modules of synaptic, immune, and metabolic proteins associated with AD severity, yet many of these changes cannot be predicted directly from transcript levels [15]. When combined with genomics, proteomics can uncover protein quantitative trait loci (pQTLs) that map genetic risk variants to their downstream protein effects, thereby connecting susceptibility loci like APOE or TREM2 to measurable protein pathways [16]. These findings emphasize that proteomics gains interpretive power when embedded in multi-omics frameworks, where it can help bridge genetic variation, RNA changes, and functional protein networks to construct a more complete model of AD pathology.

The integration of multiple omics layers, known as multi-omics integration, provides a more holistic view of disease pathology and enables researchers to deepen their understanding of the connections between different biomarkers. In fact, single layers capture only part of disease biology, while multi-omics integration links variants to downstream RNA and protein effects, prioritizes pathways, and resolves heterogeneity [17]. Common strategies include genetic-to-expression/protein mapping (eQTL/pQTL, colocalization, TWASs, Mendelian randomization), latent-factor and multi-block models (MOFA/MOFA+, iCluster, DIABLO/PLS), and network-based integration (e.g., WGCNA with protein–protein interaction graphs) [18]. Increasingly, bulk signals are anchored to single-cell and spatial maps (cell2location, NicheNet) to pinpoint cell-type-specific mechanisms [19].

For AD, these frameworks enable subtype delineation, more robust blood/CSF biomarker panels, and higher-confidence therapeutic targets [20], setting up the rationale for the integration strategies reviewed later in this paper. Here, we analyze how these integration strategies inter-connect across omics layers and how their convergence with single-cell and spatial anchoring advances mechanism-level understanding as well as diagnostic and therapeutic development.

## 2. Methods—Search Strategy and Study Selection

**Figure 1:**
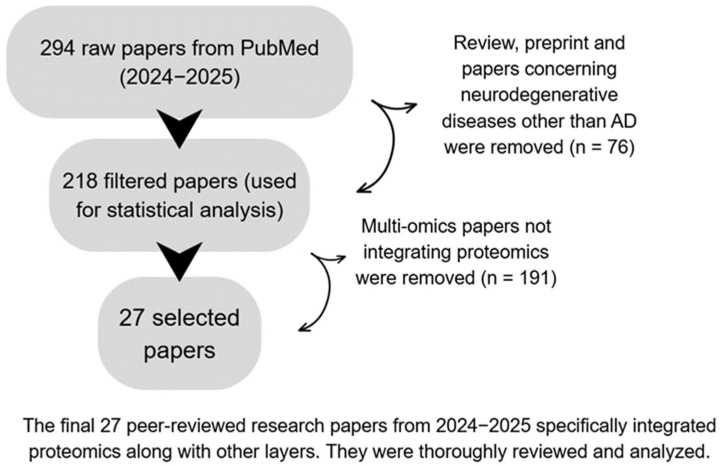
Analysis Diagram.

We conducted a structured literature screening to identify proteomics-anchored multi-omics studies in AD. Searches were performed in PubMed and restricted to 2024–2025. We have used different combination of the keywords in PubMed, such as: (“Alzheimer’s disease” [Title/Abstract] OR “Alzheimer disease” [Title/Abstract] OR “Alzheimer’s dementia” [Title/Abstract]) AND (“multi-omics” [Title/Abstract] OR “multiomics” [Title/Abstract] OR “integrative omics” [Title/Abstract] OR “systems biology” [Title/Abstract] OR “multi-omic” [Title/Abstract] OR “multi-modal omics” [Title/Abstract]). The keyword set included “Alzheimer’s disease”, “Alzheimer disease”, “Alzheimer’s dementia”, “machine learning”, “deep learning”, “AI”, “multi-omics/multiomics”, “integrative omics”, “systems biology”, “multi-omic”, “multi-modal omics”, and “single-cell/single cell”. We included peer-reviewed AD research implementing multi-omics in which proteomics was a primary analytical layer; we excluded reviews, editorials, preprints, and studies on non-AD neurodegenerative diseases. Titles/abstracts were screened, followed by full-text checks for multi-omics status and anchoring. The queries returned 792 unique records; 294 fell within 2024–2025. After full-text eligibility assessment, 26% of the 294 were excluded, leaving 218 studies contributing to the descriptive statistics; 27 proteomics-anchored studies were qualitatively synthesized. A PRISMA-style flow diagram is provided in Figure 1, and the list of 27 studies is given in Figure 1. We also include the multiple-database scopes (Web of Science Core Collection and Scopus) to check if there are more papers. This systematic review was conducted and reported in accordance with the PRISMA 2020 guidelines (Preferred Reporting Items for Systematic Reviews and Meta-Analyses). The detailed search strategy, inclusion and exclusion criteria, and study selection process followed the PRISMA 2020 checklist Supplementary Table S1. We included a summary of the original list of papers and selected papers in the Supplementary Table S2. This review has been registered through Open Science Framework with registration DOI of 10.17605/OSF.IO/PH8Q3.

## 3. Results

### 3.1. Four Major Research Pillars in Proteomics-Anchored Multi-Omics Studies

**Figure 2:**
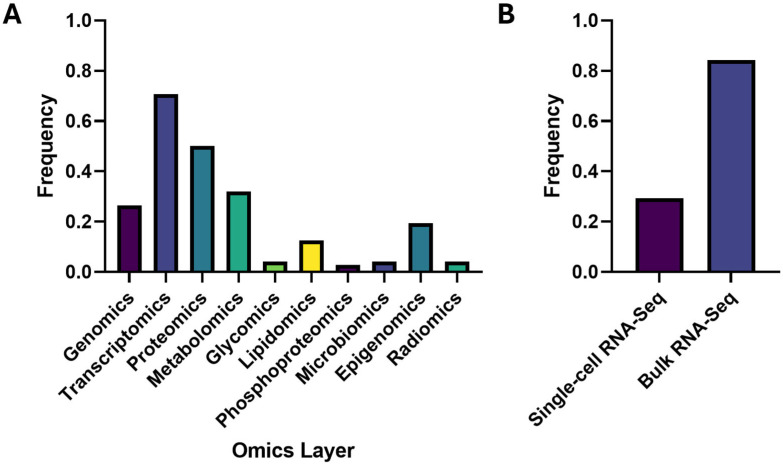
Frequency of omics layers. Panel (**A**) shows the frequency with which each omics layer is studied among the pool of 2024–2025 multi-omics research papers. Transcriptomics had the highest frequency, being studied in 70.83% of the pool, followed by proteomics with a frequency of 50.00%. Panel (**B**) shows the frequency of single-cell RNA-Seq (29.41%) and Bulk RNA-Seq (84.31%) studies among the transcriptomics studies.

**Figure 3:**
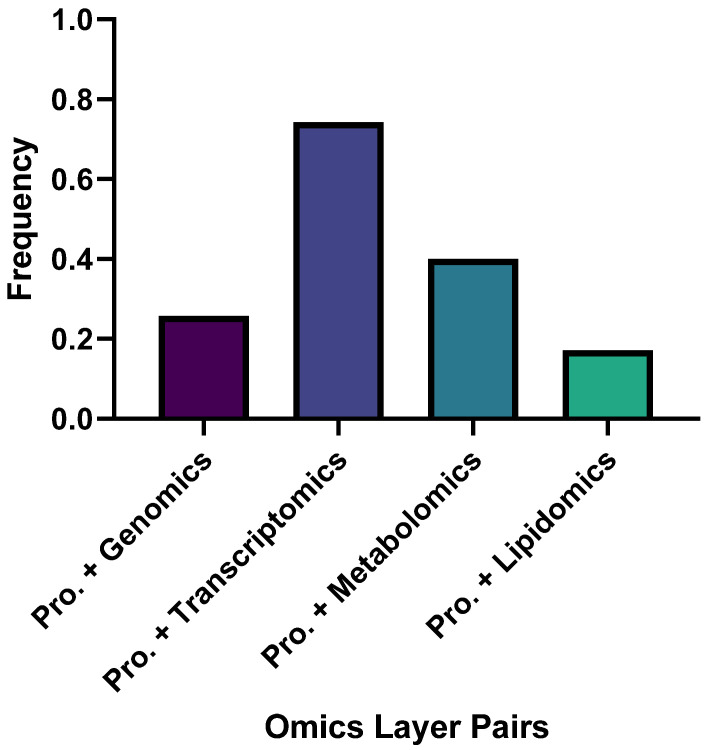
Frequency of omics layer pairs with proteomics. This shows the frequency of proteomics papers studied along with each omics layer. Proteomics was most commonly studied with transcriptomics, with a frequency of 74.29%. “Pro.” denotes paper studying proteomics; “Pro. + Genomics” denotes papers studying both proteomics and genomics. Similarly, “Pro. + Transcriptomics” denotes papers studying both proteomics and transcriptomics.

Recent proteomics-anchored multi-omics research in AD coalesces around four complementary pillars: causal and computational integration; fluid biomarkers and clinical translation; brain tissue and spatial mechanisms; and models, comorbidity and resources (Supplementary Table S3). Across these areas, several patterns recur. Proteomic readouts are often closer to phenotype than transcripts and therefore explain variance that RNA alone does not. Mitochondrial, immune–lipid, and synaptic programs repeatedly emerge as organizing axes. Finally, method choice matters: inference grounded in genetic instruments addresses directionality, whereas graph and deep learning pipelines uncover higher-order structure but require explicit biological validation. We specifically chose to analyze proteomics-centered multi-omics papers because we wanted to select a layer that was not the most or least popular one in recent years. We thus chose the proteomics layer, which was the second most studied (Figure 1). With the focus on the proteomics layer, we determined which other layer these studies tended to pair up with and found it was transcriptomics (Figure 2 and Figure 3). This informs researchers of the current trends in the field, helping them choose a relevant direction for their own work.

#### 3.1.1. Causal and Computational Integration

Zhan et al. connected AD risk alleles to downstream proteins using AD GWASs, cis-eQTLs, and cis-pQTLs and used two-sample Mendelian randomization with extensive sensitivity analyses to nominate five plasma proteins—BLNK, CD2AP, GRN, PILRA and PILRB—as putatively causal, with GRN (protective) and BLNK among the strongest candidates [21]. A second study by Zhan et al. integrated AD GWASs with blood and brain eQTLs and mQTLs and showed that mitochondrial genes contribute to risk under the influence of epigenetic and inflammatory regulation, including shared genetic control between mitochondrial loci and cytokines such as LDLR with IL-17C, ACE with IL-18, and PTPMT1 with HGF and OSM [22]. Extending causal mapping to neuroimaging, Wang et al. combined brain and plasma proteomics with imaging GWASs to implicate more than 300 proteins in brain structural traits and highlighted EGFR and TMEM106B in white-matter pathways connected to AD risk [23].

Alongside instrument-based inference, several computational frameworks integrate multi-omics with prior biology to improve prediction and interpretability. Tripathy et al. introduced GNNRAI, which aligned transcriptomic and proteomic representations using graph neural networks and a set transformer; in ROSMAP, proteomics outperformed transcriptomics for AD classification, and GNNRAI surpassed benchmarks such as MOGONET while recovering known markers and proposing candidates across lipid, mitochondrial, synaptic, and endolysosomal domains [24]. Cary et al. developed a Target Risk Score by mapping expression and protein changes onto genetic associations across thousands of genes, revealing consistent clustering of AD risk into synaptic, immune, lipid, mitochondrial, structural, and proteostasis domains and suggesting that drug pipelines may be misaligned with the biological burden emphasized by multi-omics [25]. Additional models reinforced these themes: Tu et al. combined imaging genetics and expression to propose FAM117A and ACSL1 as causal signals with immune relevance [26]; Zhang et al. introduced digID, which integrated expression, pathways and protein interactions over 133 datasets and validated G-protein signaling genes (GNAI1, GNB1, KNG1) in the human brain and 3xTg-AD mice [27]; Xie et al. proposed MoFNet to couple SNPs, RNA, and targeted peptides with prior networks and recovered subnetworks in immune signaling, protein clearance and neurotransmission including the SNARE complex, with cross-cohort replication [28]; and Yan et al. optimized network-based integration to identify transcriptional and epigenetic biomarkers of aging and AD, highlighting FOXG1, SST, and AIM2, among others [29]. Taken together, these studies show that proteomics-anchored integration can deliver both causal hypotheses and predictive structure, with convergence on mitochondrial, lipid–immune, and synaptic biology, which is illustrated through this review.

#### 3.1.2. Fluid Biomarkers and Clinical Translation

Multi-omics profiling of blood and accessible tissues is advancing toward the development of clinically relevant panels. Meng et al. combined plasma proteomics, metabolomics, and gut and saliva metagenomics across clinically stratified AD groups and linked severity to coordinated changes that included NEFL and GFAP in plasma, amino-acid, and lipid shifts and microbiome features such as Paraprevotella clara; machine learning models predicted ADAS-Cog severity from integrated features [30]. Yang et al. profiled senescence-associated secretory phenotypes and found that SASP-related proteins including AQR, ZNF587B, CRP, FN1, and SAA1 are elevated in AD, with increased serum PAGln, indicating a gut–brain axis [31]. In the EMIF-AD cohort, Gómez-Pascual et al. integrated lipidomics and proteomics to differentiate normal controls, and MCI and AD patients and to predict MCI-to-AD conversion, with oleamide and proteins such as alpha-synuclein, properdin, PI15 and JPH3 contributing to the high accuracy of the method [32]. François et al. demonstrated that saliva can capture proteomic, metabolomic, and microbial signatures correlated with disease measures, including Stratifin as a top protein linked inversely to plasma pTau181 and metabolites such as L-tyrosine and 3-chlorotyrosine associated with severity [33]. Souchet et al. translated discovery-scale blood multi-omics into the targeted B-HEALED test comprising 19 biomarkers plus age, achieving high specificity for AD dementia and near-perfect specificity when combined with amyloid modalities [34]. Across these studies, proteomic markers of axonal and astroglial injury recur in line with metabolic and microbial alterations, indicating that multi-layer assays can improve staging and conversion prediction if paired with harmonized assays and external validation.

#### 3.1.3. Brain Tissue and Spatial Mechanisms

Mechanistic resolution increases in brain tissue, particularly when molecular layers are anchored to cell types and spatial context. Zhao et al. used hMe-Seal to map 5-hydroxymethylcytosine across a large cohort and reported widespread hypo-hydroxymethylation associated with diagnosis, Aβ load, and PHFtau density; integrative analyses tied 5hmC to nearby gene expression and protein abundance and located alterations around transcription start sites and enhancers [35]. A meta-integration by Abyadeh and Kaya comparing frontal-cortex proteomes and transcriptomes found extensive protein-level alterations in mitochondrial pathways that were not mirrored at the RNA level; it was also found that high-speed-isolated extracellular vesicles carried the richest set of mitochondria-linked proteins inversely altered in AD brains, suggesting a route to restore mitochondrial function [36]. Eteleeb et al. integrated transcriptomic, proteomic, metabolomic, and lipidomic profiles with clinical and neuropathological data and identified four molecular AD profiles; one subtype displayed pronounced synaptic downregulation, astrocyte enrichment, neurotransmitter loss, and worse outcomes, all replicated across independent cohorts, with SNCA, SNAP25, GFAP, and CLU emerging as markers and single-nucleus RNA-seq revealing astrocytes and endothelial cells as key drivers [37]. Toyama et al. combined spatial proteomics, lipidomics, and imaging mass spectrometry to reveal diverse Aβ proteoforms, localized cytoskeletal and AD-dominant proteins within plaques, and white-matter lipid disruption with scattered heme deposits suggestive of microbleeds [38]. Zhang et al. bridged aging clocks, RNA modules, and proteins to nominate PBXIP1 as a hub associated with multiple neuropathologic traits and AD diagnosis across datasets [39], while Han et al. linked miR-133b to NPTX2 protein, cognition, and AD odds, suggesting a miR–protein synaptic axis [40]. Cross-species research by Yarbro et al. further showed that plaque protein accumulation largely reflects delayed protein turnover rather than transcriptional change, emphasizing the importance of proteoform dynamics [41]. Together, these studies connect chromatin, RNA, and protein layers to cell-type-specific and spatially resolved pathology; explain frequent RNA–protein divergence; and highlight targets such as PBXIP1 and NPTX2 within coherent tissue programs.

#### 3.1.4. Models, Comorbidity and Resources

Experimental systems and population-scale resources provide complementary leverage. In a time-resolved rat model of trimethyltin neurotoxicity, Zakaria et al. traced sequential transcriptomic and proteomic changes that paralleled elevations in plasma NfL and involved inflammation, apoptosis, synaptic dysfunction, and oxidative stress, with Alzheimer-related proteins such as APP, APLP1/2, PLD3, and ATP6AP2 altered at late time points [42]. The AMP-AD Diversity Initiative demonstrated the feasibility of large-scale, harmonized multi-omics across brain regions in Black American and Latin American donors, addressing a critical gap in representation and enabling cross-cohort comparisons that will be essential for generalizability and precision medicine [43]. Tate et al. showed that deletion of miR-33 in APP/PS1 mice increases ABCA1 expression and apoE lipidation, reduces plaques and gliosis, and enhances microglial migration and Aβ phagocytosis, thereby connecting lipid metabolism to immune modulation and plaque biology [44]. Nguyen et al. integrated bulk RNA, protein–protein interaction networks and single-cell RNA-seq to identify shared signatures between AD and type 2 diabetes mellitus, nominating PIP4K2A as a biomarker with protein-level support and functional links to vesicle trafficking and autophagy [45]. Lu et al. combined OrbiSIMS with metabolomics and proteomics in engineered ApoE4 cells to show broad lipid and amino-acid metabolic changes and disruptions in protein biosynthesis and RNA splicing, underscoring cell-intrinsic mechanisms relevant to AD [46]. These studies illustrate how models can expose timing and mechanism, how comorbidity-oriented analyses reveal shared pathways, and how inclusive resources create the foundation for reproducible discovery and external validation.

Cross-pillar synthesis and implications. The four pillars are tightly connected. Genetic instruments and causal mapping have identified proteins such as GRN, BLNK, EGFR and TMEM106B, which recur in predictive and network models and appear within white-matter and synaptic pathways [21,23,25,27,28]. Fluid-based panels repeatedly capture axonal and astroglial signals and metabolic readouts that echo tissue-level synaptic and mitochondrial dysregulation [30,31,32,33,34,37,38,39,40,41]. Spatial and cell-type anchoring clarifies why RNA–protein correlations are often weak and why proteomics is indispensable for resolving proteoforms and turnover [35,36,37,38,41]. Model systems reveal levers such as miR-33–ABCA1–apoE that align with human lipid–immune biology, and multi-ethnic resources ensure that signals are robust across populations [43,44]. Points of tension remain, including subtype heterogeneity, cohort and platform effects, and the need to reconcile causal estimates with correlation-based models. A practical path forward is to couple instrument-based inference with graph and deep learning models, ground both in single-cell and spatial context, and pre-specify validation in independent cohorts and targeted assays. In this framework, proteomics remains the organizing layer that links genetic risk and RNA programs to actionable protein networks for diagnosis and therapy.

## 4. Summary of the Most Common AD Mechanisms

Across the multi-omics studies discussed in Section 3, several recurring and interconnected mechanisms emerge. Proteomic readouts, being closest to the phenotype, consistently capture disease-relevant variations that often elude detection at the RNA level [45]. At the same time, integrative multi-modal analyses converge on four major axes of Alzheimer’s disease (AD) pathology—mitochondrial/metabolic dysfunction, synaptic dysfunction, neuroinflammatory and immune–lipid activation, and proteostasis impairments—whose interplay helps explain the frequent discordance between transcript and protein measures and the heterogeneity observed across cohorts [45,46].

### 4.1. Mitochondrial Dysfunction and Metabolic Dysregulation

Deep proteomic maps of AD cortex consistently show a downregulation of oxidative phosphorylation and related metabolic pathways at the protein level, with much weaker or absent changes at the bulk RNA level [46]. This indicates that translational and post-translational mechanisms contribute substantially to metabolic pathology in AD. Moreover, fluid and CSF proteomic studies across the disease spectrum link metabolic and glial programs to imaging-defined pathology and disease progression, reinforcing that mitochondria-centered biology is both causally proximal (implicated early in disease etiology) and phenotypically proximal (closely tied to clinical and pathological manifestations) [45,46]. In sum, impaired energy metabolism and mitochondrial function emerge as robust protein-level signals in AD that may not be apparent from transcriptomics alone.

### 4.2. Synaptic Dysfunction

Synapse-related pathways emerge as a common theme across multiple integrative approaches. Proteomic profiling of AD brains identifies co-expression modules enriched for proteins involved in neurotransmission and synaptic vesicle cycling, and these modules correlate with clinical severity and cognitive decline [47]. Complementing this, longitudinal CSF studies have uncovered synapse-derived proteins—particularly the neuronal pentraxins (e.g., NPTX2)—as strong prognostic biomarkers of decline. Notably, lower baseline levels of CSF NPTX2 are associated with earlier progression from normal cognition to mild cognitive impairment (MCI), even after accounting for canonical amyloid and tau biomarkers [48]. Together, these data connect synaptic failure in AD to both upstream drivers (e.g., genetic or regulatory factors that influence synaptic maintenance) and downstream outcomes measurable in biofluids. In other words, synaptic integrity appears to be a key nexus linking molecular pathology to future cognitive trajectories [10].

### 4.3. Neuroinflammation and Immune–Lipid Activation

Converging evidence from genetics and proteomics implicates chronic neuroinflammation and lipid dysregulation as central features of AD. Large genome-wide association studies have highlighted numerous AD risk loci related to the innate immune system and cholesterol/lipid metabolism, suggesting that these biological domains are heavily burdened in the disease [49]. Proteomic analyses complement this by showing robust activation of astroglial and complement pathways in both brain tissue and CSF across disease stages [50]. For example, quantitative proteomics of CSF have revealed modules of complement proteins and other immune-related factors that mirror changes observed in AD brains [50]. In addition, recent evaluations of blood-based biomarker panels have confirmed that markers of astroglial activation (e.g., GFAP) and axonal injury (e.g., NfL), together with phosphorylated tau, significantly improve diagnostic and staging accuracy when incorporated into multi-analyte frameworks [41]. These convergent signals across tissues and biofluids underscore that immune activation and lipid imbalances are not peripheral phenomena but rather core, systems-level features of AD pathology [49,50].

### 4.4. Proteostasis and Delayed Protein Turnover

Multi-species proteomic studies (in human brains and AD model organisms) indicate that the accumulation of amyloid-associated proteins in plaques often reflects impaired protein turnover rather than increased production at the transcript level [51]. In other words, many proteins found enriched in amyloid plaques are not up-regulated in mRNA, suggesting that reduced clearance or degradation (proteostasis failure) underlies their accumulation. This helps explain frequent RNA–protein mismatches in AD and highlights the importance of measuring protein isoforms and degradation pathways directly [51]. Furthermore, spatial and imaging mass spectrometry approaches have provided region-specific insights into proteostasis defects. Matrix-assisted laser desorption/ionization imaging (MALDI-MSI) of AD brain tissue reveals heterogeneous Aβ proteoforms within plaque-rich regions—shorter Aβ peptides (e.g., Aβ_1–36/38/40) tend to localize around cerebral vasculature, whereas longer forms (Aβ_1–42/43) localize in parenchymal plaques [52]. These studies also show accompanying disruptions in lipid composition between white-matter and gray-matter regions, implicating demyelination and membrane lipid alterations as downstream consequences of local proteostasis failure [53]. In summary, impaired protein clearance (whether through autophagy-lysosomal pathways or other proteolytic systems) contributes to the buildup of toxic proteins and lipids in a region-dependent manner in AD.

### 4.5. Tau Phosphorylation and Proteoform-Specific Pathology

At the level of specific peptides and proteoforms, tau protein pathobiology illustrates the close integration of post-translational modification with disease phenotype. Emerging evidence indicates that the phosphorylation occupancy of tau protein at certain sites provides a more precise readout of AD pathology than total levels of tau protein or the traditional p-tau_181 marker. In particular, CSF tau phosphorylation at threonine-217 and threonine-205 has been shown to correlate more strongly with amyloid and tau PET measures, outperforming p-tau_181 as biomarkers of plaque and tangle burden [54]. The biochemical specificity of tau changes is further underscored by antibody studies: the widely used monoclonal antibody AT8 (a key histopathological tool) recognizes tau only when it is phosphorylated at serine 202 and threonine 205 [55]. This phospho-epitope (S202/T205) is leveraged in both neuropathology and proteomic workflows to identify disease-associated tau conformations. Functional studies have also demonstrated that phosphomimetic mutations at these AT8 sites (S202E/T205E in tau) can enhance tau aggregation and toxicity, linking specific tau modifications to neuronal dysfunction [56,57]. Finally, large-scale proteomic analyses of CSF and brain place these tau changes in context: tau proteoform alterations co-cluster with glial, synaptic, and metabolic protein modules that progressively shift in tandem with increasing amyloid plaque and neurofibrillary tangle loads [46,50]. In essence, pathologic tau phosphorylation is both a marker and a mediator of AD-related network changes, bridging upstream molecular events and downstream neurodegenerative processes.

Integrated Perspective: The four axes of dysfunction—mitochondrial energy failure, synaptic degeneration, immune–lipid dysregulation, and impaired proteostasis—form an interdependent network in AD. These processes feed into one another and together help explain why mRNA–protein correlations are often weak in diseased brains: cell-type composition shifts, regulatory (epigenetic/translational) changes, and protein turnover dynamics all modulate protein abundances and proteoform profiles beyond what transcripts alone can predict [46,51]. Importantly, agreement on these key mechanisms has emerged from diverse lines of evidence, including human genetics, causality-focused analyses (e.g., Mendelian randomization and quantitative trait loci studies), deep learning and network models, spatially resolved omics, and fluid biomarkers. This convergence across independent approaches increases confidence that the highlighted axes are mechanistically connected drivers of AD rather than unrelated observations. It also motivates future research to design proteomics-anchored, multi-layer studies that explicitly test these mechanisms across cohorts. Validating these pathways in a pre-specified, multi-omics framework (for example, linking genetic risk to proteomic changes to clinical outcomes) will be crucial for translating these insights into therapeutic and biomarker development [10,55]. In summary, the common mechanisms identified serve as a roadmap for understanding AD biology and underscore the added value of proteomics in illuminating disease processes that might be overlooked by either genomics or transcriptomics alone.

## 5. Discussion and Future Perspectives

Anchoring this section to the premise in the Introduction, we focus on how proteomics can help close the RNA–protein gap by moving from description to mechanism. Recent brain proteome maps highlight pathway-level shifts—especially in mitochondrial and energy metabolism and in synaptic programmes—that are not mirrored at bulk RNA levels, indicating that regulation downstream of transcripts is central to AD pathophysiology [36]. This is consistent with work showing that steady-state mRNA–protein correlations often explain only a minority of protein variance (≈40%), underscoring the need to interrogate regulation beyond transcripts [58]. Synthesizing Section 4, a testable network emerges: mitochondrial energy failure and impaired proteostasis plausibly precede and mediate synaptic loss, while immune–lipid activation (for example, complement and astroglial programmes) amplifies both turnover defects and synaptic vulnerability; τ proteoform changes (for example, shifts in phosphorylation occupancy) track these axes and connect molecular states to clinical trajectories [59]. This integrated view helps explain why protein-level effects can persist when RNA signals are weak or absent and suggests specific cross-axis couplings that can be tested across tissues and biofluids.

To move from this conceptual model to mechanism, future studies should directly separate protein synthesis from degradation in the same samples where RNA–protein discordance is observed. Examples include ribosome profiling to estimate translational efficiency, time-resolved p/d-SILAC (optionally combined with multiplexed TMT) to quantify production and clearance rates, and longitudinal sampling across matched brain–CSF–plasma to relate each kinetic component to imaging and clinical endpoints. Convergence across modalities should be evaluated using pre-registered, cross-cohort analyses that pre-specify primary biological axes (synaptic, immune–lipid, mitochondrial) and test whether protein-level effects persist after adjustment for single-cell-inferred cell-type composition, thereby distinguishing true post-transcriptional biology from mixture artefacts [59].

On the analysis side, the goal is not to introduce yet another tool catalogue, but to promote standards that turn integrative models into testable biology. Mechanism-aware integrators—factor and network frameworks such as MOFA+, DIABLO, and WGCNA—yield latent axes or modules shared across modalities that can be functionally tested, avoiding purely black-box outputs [59,60]. Claims of discovery should be supported by robust generalization, including leakage-safe preprocessing, nested cross-validation, external-cohort validation, and transparent reporting of hyperparameters and analysis provenance in high-dimensional settings. Interpretability should be treated as a core deliverable: any performant predictor should be paired with stable feature attribution and perturbation tests so that nominated genes or proteins become falsifiable hypotheses for experimental follow-up. For prioritization, triangulation toward upstream drivers is critical: linking protein modules to inherited variation through pQTL/eQTL-based colocalization or Mendelian randomization can enrich for candidates that lie closer to causal mechanisms rather than downstream correlates, and are therefore more promising as mechanistic and therapeutic targets [60].

Heterogeneity in AD further argues for designs that move beyond cohort-average effects and work across ancestries and molecular subtypes. Multi-omics studies in under-represented groups suggest broadly conserved AD pathways but quantitative differences in module amplitudes and biomarker levels, indicating that both discovery and validation sets should be ancestrally diverse and that any biomarker panel should report subgroup-specific calibration and decision-curve utility [43]. Proteome-anchored subtype labels should be prospectively linked to outcomes and treatment selection, rather than remaining purely descriptive, with harmonized covariate capture (for example, age, post-mortem interval, RNA integrity number, batch) and shared pipelines to enable cross-site replication [37].

Building on this synthesis, we therefore propose a set of focused future directions that move beyond listing pathways to outlining concrete next steps for the field. (i) Pre-registered cross-axis mediation tests: quantify whether immune–lipid modules statistically mediate the effect of mitochondrial protein deficits on synaptic protein decline, with success predefined by significant indirect paths after multiple-testing correction [59,60]. (ii) Kinetic dissection in matched brain–CSF–plasma: estimate pathway-specific translation and degradation rates and nominate drug-responsive kinetic nodes (for example, degradation bottlenecks) as interventional targets [36,58]. (iii) Proteoform-resolved endpoints: track phospho- and ubiquitino-proteoforms that co-shift with the Section 4 modules and that outperform transcript surrogates on prognostic or theragnostic utility [60]. (iv) Subtype-aware validation: prospectively test whether astroglia-dominant versus synapse-dominant subtypes differentially benefit from glia-targeted versus synaptic-support strategies, with stratified power and pre-specified decision thresholds [37,43]. (v) Benchmarked machine-learning pipelines: release fixed train/test splits, leakage checks, nested cross-validation plans, and mandatory interpretability reports before making biomarker-readiness claims.

Translational progress, finally, hinges on a pragmatic agenda aligned with a proteomics-first view. Key elements include adequately powered and harmonized longitudinal brain–CSF–plasma collections with pre-specified endpoints; FAIR-style openness with shared raw and processed matrices plus code; routine inclusion of dynamics and proteoforms to surface drug-responsive kinetic nodes; and community benchmarks for integrative machine learning with fixed splits, leakage checks, nested cross-validation, and interpretability as a standard requirement [60]. Together, these steps maintain the proteomics-anchored stance of this review but turn it into a practical roadmap for mechanism-based precision medicine in AD, linking genetic susceptibility and RNA programmes to functional protein modules and clinically deployable biomarker panels that extend beyond Aβ and tau.

## 6. Conclusions

This review synthesizes recent (2024–2025) multi-omics research in Alzheimer’s disease (AD) within a proteomics-anchored framework. By screening over 400 studies and closely examining more than 200, we delineate core molecular mechanisms, integration strategies, and analytic approaches that currently define the field.

Across these studies, proteomics emerges as a distinctive layer: genomics and transcriptomics capture upstream risk and regulatory variation, whereas proteomics measures the proximate effectors of cellular dysfunction. Well-documented transcript–protein discrepancies highlight layers of translational regulation and protein turnover that are invisible to RNA-based approaches, underscoring why proteomics-enabled multi-omics integration is essential for linking variants, pathways, and phenotypes in AD.

The urgency now is to turn this molecular understanding into actionable precision. Multi-omics data already reveal reproducible axes—such as synaptic dysfunction, neuroinflammation, mitochondrial impairment, and disrupted proteostasis—and show that these signals vary across spatial, temporal, and cell-type contexts, as well as across ancestries and molecular subtypes. To prevent these insights from remaining purely descriptive, the next phase must prioritize adequately powered and harmonized cohorts, transparent and reproducible integration pipelines, and rigorous validation of causal mechanisms and candidate biomarkers in experimental systems and clinical settings.

In sum, proteomics embedded within well-designed multi-omics studies offers not just additional data, but a critical dimension of insight into AD biology at precisely the moment when new technologies, expanding cohorts, and increasing disease burden demand mechanism-based solutions. Realizing this promise will depend on using proteomics-informed multi-omics to identify meaningful patient subtypes, refine diagnostics, and guide mechanism-based therapies, thereby moving beyond Aβ- and tau-centric views toward a more complete and clinically actionable model of AD.

## Data Availability

No new data were created or analyzed in this study.

## Supplementary Materials

The following supporting information can be downloaded at: https://www.mdpi.com/article/10.3390/neurolint17120197/s1, Table S1: PRISMA 2020 Checklist; Table S2: Overview of all identified studies and those included after screening; Table S3: Quantitative summary of multi-omics studies in Alzheimer’s disease.

## Abbreviations

The following abbreviations are used in this manuscript:

AD	Alzheimer’s disease
NDs	Neurodegenerative diseases
MCI	Mild cognitive impairment
CN	Cognitively normal
NC	Normal control
ADAS-Cog	Alzheimer’s Disease Assessment Scale–Cognitive Subscale
BMI	Body mass index
ADNI	Alzheimer’s Disease Neuroimaging Initiative
ROSMAP/ROS/MAP	Religious Orders Study/Memory and Aging Project
MSDD	Mount Sinai Brain Bank
ARIC	Atherosclerosis Risk in Communities
EMIF-AD	European Medical Information Framework-Alzheimer’s Disease
AMP-AD	Accelerating Medicines Partnership-Alzheimer’s Disease
ADRC	Alzheimer’s Disease Research Center
SAND	SAND cohort (study name as cited)
ACC	Anterior cingulate cortex
PCC	Posterior cingulate cortex
PHG	Parahippocampal gyrus
FP	Frontal pole
IFG	Inferior frontal gyrus
STG	Superior temporal gyrus
TCX	Temporal cortex
DLPFC	Dorsolateral prefrontal cortex
BM36	Brodmann area 36
CAA	Cerebral amyloid angiopathy
Aβ	Amyloid-β
pTau181	Phosphorylated tau at threonine 181
PHFtau	Paired helical filament tau
APP	Amyloid precursor protein
APOE (ε4)	Apolipoprotein E (ε4 allele)
PSEN1/PSEN2	Presenilin-1/Presenilin-2
TREM2	Triggering receptor expressed on myeloid cells 2
MAPT	Microtubule-associated protein tau
GFAP	Glial fibrillary acidic protein
NEFL	Neurofilament light
NPTX2	Neuronal pentraxin 2
ABCA1	ATP-binding cassette transporter A1
PBXIP1	Pre-B-cell leukemia homeobox interacting protein 1
EGFR	Epidermal growth factor receptor
TMEM106B	Transmembrane protein 106B
HLA-B	Human leukocyte antigen-B
CLU	Clusterin
LDLR	Low-density lipoprotein receptor
ACE	Angiotensin-converting enzyme
PTPMT1	Protein-tyrosine phosphatase mitochondrial 1
IL-17C/IL-18	Interleukin-17C/Interleukin-18
HGF	Hepatocyte growth factor
OSM	Oncostatin M
GABA	Gamma-aminobutyric acid
DNA	Deoxyribonucleic acid
RNA	Ribonucleic acid
mRNA	Messenger RNA
ncRNAs	Non-coding RNAs
miRNA/miRNAs	MicroRNA(s) (e.g., miR-33, miR-133b)
piRNAs	PIWI-interacting RNAs
siRNAs	Small interfering RNAs
circRNAs	Circular RNAs
CpG	Cytosine–phosphate–guanine dinucleotide
5hmC	5-hydroxymethylcytosine
GWAS	Genome-wide association study
QTL	Quantitative trait locus
eQTL/pQTL/mQTL	Expression/protein/methylation QTL
TWAS	Transcriptome-wide association study
MR	Mendelian randomization
MR-Egger	MR-Egger regression
MR-PRESSO	Mendelian Randomization Pleiotropy RESidual Sum and Outlier
DIABLO	Data Integration Analysis for Biomarker discovery using Latent cOmponents
PLS	Partial least squares
MOFA/MOFA+	Multi-Omics Factor Analysis/MOFA Plus
iCluster	Integrative clustering framework
WGCNA	Weighted gene co-expression network analysis
PPI	Protein–protein interaction
ATN	Amyloid/Tau/Neurodegeneration (biomarker framework)
RNA-seq	RNA sequencing
ChIP-seq	Chromatin immunoprecipitation sequencing
hMe-Seal	Hydroxymethylation selective chemical labeling (for 5hmC)
LC-MS/MS	Ultra-performance LC–MS/MS
DDA/DIA	Data-dependent/data-independent acquisition
SRM/PRM	Selected/parallel reaction monitoring
MALDI-MSI	Matrix-assisted laser desorption/ionization mass spectrometry imaging
TMT-MS/TMT	Tandem mass tag mass spectrometry/Tandem mass tags
STORM	Stochastic Optical Reconstruction Microscopy
OrbiSIMS	Orbitrap Secondary Ion Mass Spectrometry
CRISPR/Cas9	Clustered Regularly Interspaced Short Palindromic Repeats/CRISPR-associated protein 9
CSF	Cerebrospinal fluid
Olink	Olink proximity-extension assay platform
SomaScan	SomaLogic aptamer-based proteomics platform
GNNRAI	Graph Neural Network-derived Representation Alignment and Integration
MOGONET	Multi-Omics Graph cOnvolutional NETwork
MoFNet	(Model name; multi-omics network)
SNARE	Soluble N-ethylmaleimide-sensitive factor Attachment protein REceptor (complex)
EV/HS-EVs	Extracellular vesicle/High-speed-isolated EVs
TRS	Target Risk Score
AAV-AD	Adeno-associated virus-based AD model
3xTg-AD	Triple-transgenic Alzheimer’s disease mouse
T2DM	Type 2 diabetes mellitus
BA/LA	Black American/Latin American
ML	Machine learning
SVM	Support vector machine
RF	Random forest
k-NN	k-nearest neighbor
XGBoost	eXtreme Gradient Boosting
