# Supplementary material for: Systematic Review: Proteomics-Driven Multi-Omics Integration for Alzheimer’s Disease Pathology and Precision Medicine
Source: Neurol Int. 2025 Dec 2;17(12):197. doi: 10.3390/neurolint17120197 (PMC12736289; doi:10.3390/neurolint17120197)
Supplement: Supplementary file 1 [file neurolint-17-00197-s001.zip › Supplementary Table S3.pdf]

Supplementary Table S3. Quantitative summary of multi-omics studies in Alzheimer's disease.

Study (short title)	Pillar	Omics layers	Tissue / modality (human / animal)	Method class	Main axes / pathway focus	Replication / convergence status
An integrative systems-biology approach defines mechanisms of AD neurodegeneration	Causal/Computational (3.1)	G, T(single & bulk), P, M, L, Phospho	Aging human cortex; laser-captured pyramidal neurons; *Drosophila* brain	Multi-layer network integration	Synaptic, mitochondrial/energy, immune–lipid, proteostasis modules	Multi-cohort human + cross-species convergence
Multi-omics analysis of druggable genes to facilitate AD therapy	Causal/Computational (3.1)	G, T(bulk)	Human brain tissue; peripheral blood	Multi-cohort ML + network integration	Druggable gene modules across synaptic, immune, mitochondrial pathways	Multi-cohort human; cross-cohort convergence
Biomarker identification for AD via MR and proteomics	Causal/Computational + Fluid (3.1/3.2)	G, P	Plasma, blood, brain regions (European & African ancestry)	Two-sample MR + proteomic integration	Immune–lipid, complement and metabolic proteins as causal biomarkers	Very large multi-cohort; cross-ancestry convergence
Expansion of peripheral cytotoxic $CD4^+$ T cells in AD	Fluid biomarker (3.2)	G, T(single & bulk), P, M, Epi	Human PBMCs / peripheral blood	Multi-omics profiling + network/association	Peripheral cytotoxic T-cell activation; immune–inflammatory axes	Single-cohort; internal multi-omics convergence
Multi-omics analysis for cell-type-specific druggable targets in AD	Causal/Computational (3.1)	G, T(bulk), Epi	Human & mouse PBMCs	scFEA-based neural network + integration	Cell-type-specific metabolic and signaling pathways in immune cells	Cross-species (human + mouse); small n per organism

Brain 5-hmC alterations associated with AD neuropathology	Causal/Computational (3.1)	G, T(bulk), P, Epi (5-hmC)	Human dorsolateral prefrontal cortex (community cohort)	Elastic net regression + multi-omics association	Epigenetic regulation of synaptic, immune and metabolic pathways	Large single cohort (~1050); internal cross-omics replication
Deciphering novel mitochondrial signatures across AD and glioblastoma	Causal/Computational (3.1)	T(single & bulk), Epi	Human DLPFC nuclei; glioblastoma tumor tissue	Classical ML classifiers + pathway/network analysis	Mitochondrial/energy metabolism; oligodendrocyte/glial pathways	Cross-disease (AD + GBM) multi-cohort convergence
Human and mouse proteomics reveal shared pathways and delayed turnover	Causal/Computational (3.1)	P, Phospho	Human and mouse brain	Quantitative proteomics; turnover modeling	Amyloid-related proteostasis; synaptic and metabolic pathways	Cross-species convergence (human + mouse)
Aspirin-associated reduced AD risk: multi-omics analysis	Causal/Computational (3.1)	G, T(bulk)	Post-mortem brain; large clinical cohorts	TWAS, signature reversal, regression	Inflammatory and metabolic pathways linked to aspirin exposure	Multi-cohort TWAS; >1M clinical validation set
MINDSETS: multi-omics + neuroimaging for dementia subtyping	Causal/Computational (3.1)	G, P, Rad	Neuroimaging + blood/brain-related data	3D U-net, ensemble classifiers, deep feature generation	Imaging–omics subtypes; neurodegeneration and connectivity pathways	1k+ participants; internal train/validation splits
APOE genotype and biological age impact inter-omic associations	Causal/Computational (3.1)	P, M	Human plasma	Correlation networks; statistical modeling	Bioenergetic and metabolic modules modulated by APOE, sex, age	Single large cohort (~2200); stratified by genotype/age

Integrated multi-omics of synaptosomes reveals synapse-centered targets	Causal/Computational (3.1)	T(bulk), P	Human frontal cortex synaptosomes	DIABLO multi-omics integration	Synaptic modules; vesicle trafficking; immune–lipid cross-talk	Single-cohort AD vs control; module-level replication across omics
Interaction of genetic variants and methylation regulating expression in AD	Causal/Computational (3.1)	G, T(bulk), Epi	Human DLPFC and parahippocampal gyrus	Regression, likelihood ratio tests, LD-based clumping	Genetic–epigenetic regulation of differentiation and immune pathways	Single-cohort (~400); cross-omics regulatory convergence
Multiomics reveals mechanisms linking structural covariance to NPS	Causal/Computational (3.1)	G, T(bulk), P, Rad	Neuroimaging + peripheral blood	Unsupervised clustering, consensus clustering, GWAS, GSEA	Macro-scale network dysfunction; neuropsychiatric symptom subtypes	600+ adults; internal subtype replication/ stability
AD-GCN: graph convolutional network integrating multi-omics	Causal/Computational (3.1)	G, Epi	Human peripheral blood	Feature selection + GCN / GraphSAGE	Multi-omics risk score; immune and regulatory pathways	Single-cohort (~589); internal CV; no external cohort
M3NetFlow: multi-scale multi-hop graph AI for integrative multi-omics	Causal/Computational (3.1)	T(single-cell), P	PBMCs, CSF, plasma	Graph-based AI; RF classifier	Immune, inflammatory and synaptic axes across compartments	Small cohort (~67); cross-compartment convergence
Integrative multiomics reveals common endotypes in familial AD	Causal/Computational (3.1)	T(bulk), P	Post-mortem brain from PSEN1/2/APP mutation carriers	Multi-omics clustering / endotyping	Synaptic, immune–lipid, mitochondrial endotypes	Single familial cohort (~115); internal subtype robustness

Multiomics from AD brains and MSC-derived EVs: mitochondrial proteostasis	Causal/Computational + Fluid (3.1/3.2)	T(bulk), P	Human brain; peripheral blood; plasma EVs	ML classifiers (Lasso, RF, SVM, NN)	Mitochondrial proteostasis; EV cargo–brain axis	Moderate n (~63); train/validation within cohort
Chemiluminescence signature arrays + ML for serum AD diagnosis	Fluid biomarker (3.2)	P	Human serum	RF, SVM, neural network	Multi-protein chemiluminescence panel; immune & inflammatory signatures	Single-cohort (n≈112); internal train/test split
Blood biomarker profiles in young-onset neurocognitive disorders	Fluid biomarker (3.2)	G, P	Human blood	Information-theoretic model selection	Serum biomarker patterns for EO-AD vs other YOND	Single cohort (n≈65); exploratory
Integrative sc and plasma RNA identifies biomarkers for early AD	Fluid biomarker (3.2)	T(single-cell), T(bulk plasma)	Human prefrontal cortex; plasma	Lasso, RF, SVM	Early, non-invasive plasma RNA signatures linked to brain cell states	Discovery (n=189) + validation (n=70) cohorts
Prediction of MCI using blood multi-omics data	Fluid biomarker (3.2)	T(bulk), M	Human blood (KoGES cohort)	Lasso, elastic net, RF, SVM, GBM	Multi-omics panel for MCI prediction; metabolic and inflammatory axes	Large single cohort (n=1410); internal CV
Multi-omics analysis reveals key factors in AD severity	Fluid biomarker (3.2)	T(bulk), P, M, L, Micro	Blood plasma, CSF, fecal samples	RF, Lasso, SVM	Severity-associated modules across plasma, CSF, gut–brain axis	Single AD cohort (n≈141); cross-compartment convergence

Systematic characterization of gut metabolites –GPCRome in AD	Fluid biomarker (3.2)	T(bulk), M	Fecal samples; PBMCs	RF classifier; ROC analysis	Gut microbial metabolites – GPCR signaling; immune–metabolic pathways	Single cohort (n≈172); internal train/test
Multi-omics analyses of gut microbiota–metabolites –brain–cognition	Fluid biomarker (3.2)	M, Micro, Rad	Fecal samples; brain imaging	Multi-omics correlation / pathway models	Gut–brain cognition pathways; metabolic & inflammatory axes	Single human cohort; cross-modality convergence
Time-restricted feeding mitigates AD via B. pseudolongum–propionate	Other (gut–brain intervention)	T(bulk), M, Micro	Mouse brain (hippocampus /cortex); fecal microbiota	Multi-omics correlation / mediation	Gut microbiota–propionate–FFAR3 axis; neuroinflammation	Single mouse model; no human replication
Multi-omics gut–microbiome –brain diet (modified Mediterranean diet)	Other (diet / gut–brain)	Metabolomics, Micro	Human stool, plasma, serum; mouse tissues (hippocampus, serum, cecum)	RF, sparse PLS-DA	Diet–microbiome–metabolite–brain function axis	Human RCT + mouse validation; cross-species convergence
Brain high-throughput multi-omics reveals molecular heterogeneity in AD	Causal/Computational (3.1)	T(bulk), P, M, L, Epi	Multi-region human brain (prefrontal, temporal, cerebellum, etc.)	Network-based clustering; MOFA	Molecular subtypes across synaptic, immune–lipid, mitochondrial axes	Multi-cohort, multi-region convergence
Single-cell transcriptomes and multiscale networks in AD	Causal/Computational (3.1)	T(single-cell)	Human prefrontal cortex	WGCNA, multiscale network modeling	Cell-type-specific synaptic, immune, glial networks	Single large scRNA-seq dataset; internal network robustness

Mitochondrial dysfunction genes, methylation, cytokines: MR study	Causal/Computational (3.1)	G, T(bulk), P, Epi	Cortical regions, blood (summary-level data)	Two-sample & multivariable MR; colocalization	Mitochondrial, inflammatory, epigenetic pathways causally linked to AD	Very large GWAS-scale samples; cross-dataset convergence
A comprehensive multi-omics analysis reveals signatures to predict AD	Causal/Computational + Fluid (3.1/3.2)	T(bulk), P, M	Blood plasma & serum; post-mortem cortical tissue	RF, SVM, PCA	Predictive signatures spanning plasma and brain; synaptic & immune–lipid	Large cohorts (~830); train/validation; cross-tissue convergence
A multi-omics study of senescence-associated secretory phenotypes in AD	Causal/Computational (3.1)	T(bulk), P	Human hippocampus, temporal cortex	PLS-DA; WGCNA	SASP-related inflammatory and proteostasis pathways	Moderate cohort (~69); internal module replication
APOE transcript / functional elements associated with AD	Causal/Computational (3.1)	G, T(single & bulk), Epi	Post-mortem brain (multi-ancestry)	RF, conditional random field, motif analysis	APOE-related regulatory elements; lipid / immune pathways	>1000 brains; European + African ancestry; cross-ancestry signals
Miss-SNF: multimodal patient similarity integration (not AD-specific)	Methodological (Other)	T(bulk), P, M	Breast & pancreatic tumor tissues	Similarity network fusion; miss-SNF	General multi-omics patient stratification	Multi-cancer cohorts; methodological generalization
snCED-seq: cryogenic nuclei dissociation for snRNA-seq (FFPE)	Methodological (Other)	T(single-nucleus)	Human colorectal & lung cancers; mouse brain	Single-nucleus RNA-seq pipeline	Technical: preserving transcriptional signatures in FFPE tissues	Multiple datasets; technical validation
